# Simulation of Abnormal Grain Growth Using the Cellular Automaton Method

**DOI:** 10.3390/ma17010138

**Published:** 2023-12-27

**Authors:** Kenji Murata, Chihiro Fukui, Fei Sun, Ta-Te Chen, Yoshitaka Adachi

**Affiliations:** 1 Engineering Steel Research Sect., Corporate Research & Development Center, Daido Steel Co., Ltd., 30, Daido-cho 2-chome, Minami-ku, Nagoya 457-8545, Japan; 2 Department of Material Design Innovation Engineering, Nagoya University, Furo-cho, Chikusa-ku, Nagoya 464-8603, Japanadachi.yoshitaka@material.nagoya-u.ac.jp (Y.A.)

**Keywords:** grain growth, cellular automaton, carburizing

## Abstract

The abnormal grain growth of steel, which is occurs during carburization, adversely affects properties such as heat treatment deformation and fatigue strength. This study aimed to control abnormal grain growth by controlling the materials and processes. Thus, it was necessary to investigate the effects of microstructure, precipitation, and heat treatment conditions on abnormal grain growth. We simulated abnormal grain growth using the cellular automaton (CA) method. The simulations focused on the grain boundary anisotropy and dispersion of precipitates. We considered the effect of grain boundary misorientation on boundary energy and mobility. The dispersion state of the precipitates and its pinning effect were considered, and grain growth simulations were performed. The results showed that the CA simulation reproduced abnormal grain growth by emphasizing the grain boundary mobility and the influence of the dispersion state of the precipitate on the occurrence of abnormal grain growth. The study findings show that the CA method is a potential technique for the prediction of abnormal grain growth.

## 1. Introduction

Steel gears are widely used in the power transmission components of automobiles. Considering that such gears are required to exhibit high surface hardness and high toughness, the surfaces of alloy steels, such as JIS-SCR420, are carburized. To achieve carbon neutrality, it is important to reduce CO_2_ emissions during automobile manufacturing. Hot forging, a metal-shaping process employed during the manufacture of automotive gears, frequently leads to CO_2_ emissions. Contrarily, cold forging reduces CO_2_ emissions during manufacturing. However, the strain produced during cold forging causes the abnormal growth of austenite grains during carburization, affecting the heat treatment deformation and fatigue strength of the gears.

Thus, the prediction of abnormal grain growth is industrially important. However, it is difficult to experimentally estimate the effect of material factors, such as precipitate amount, precipitate dispersion, and initial austenite grains, and observe abnormal grain growth during heat treatment. Thus, we developed a cellular automaton (CA) simulation method to reproduce abnormal grain growth.

Several methods for simulating grain growth exist, such as the Monte Carlo [1,2,3,4], CA [5,6,7,8], and phase-field methods [9,10]. These methods have been employed to perform simulations for normal and abnormal grain growth. For example, Hayakawa and Szpunar stated that abnormal grain growth occurs when the misorientation of the grain boundary affects the grain boundary energy and mobility [11,12]. They considered the misorientation of grain boundaries and employed the Monte Carlo method to simulate abnormal grain growth. Ye et al. [13] simulated abnormal grain growth by introducing the anisotropy of grain boundaries into the CA method.

The pinning effect of precipitates is known to play an important role in abnormal grain growth. Considering practical materials, such precipitates can exist in a state where the precipitates are biased in the matrix phase because of the introduction of elements, such as solidification segregation and carburization. For example, Kinoshita and Ohno simulated abnormal grain growth using the phase-field method by introducing the effects of the C content due to carburization and the pinning effect of NbC. They considered a situation where the precipitation state of NbC was dependent on its location, i.e., the dispersion state of NbC [14]. However, to the best of our knowledge, no simulation study has considered the anisotropy of grain boundaries and the dispersion state of precipitates.

In a previous CA study, a grain orientation was assigned with a grain number [15]. This simplicity caused a different misorientation distribution from that described in the methods section of the present report. This uncertainty should be avoided as much as possible.

Thus, this study simultaneously highlighted the effects of grain boundary mobility and precipitate dispersion on abnormal grain growth using the CA method, where a crystallographic orientation was assigned to a grain rather than a grain number.

The distinctive feature of the CA method, as opposed to the phase-field method, lies in its discrete treatment of interfaces. It allows for the physical handling of “grain boundary energy”. These characteristics make it intuitively understandable and enable the incorporation of physics into models at a lower computational cost. Based on these features, the CA method was chosen for adoption in this case.

## 2. Methods

To investigate the grain growth behavior of austenite during heat treatment, we constructed a grain growth model using the two-dimensional (2D) CA method as follows.

First, the general theory of grain growth was incorporated into the CA method. According to the general theory of grain growth, the driving force of grain growth per unit volume was calculated using the following equation in the case of simulation:

(1) $$∆G=\frac{\sigma \left[x,y\right]}{R_{gb}\left[x,y\right]},$$

where *R_gb_* is the radius of curvature, *σ* is the grain boundary energy, and *x* and *y* are the 2D coordinates of the cell. The grain boundary mobility, *M*, was dependent on temperature and expressed by Equation (as follows:

(2) $$M\left[x,y\right]=M_{0}\exp\left(-\frac{Q}{RT}\right),$$

where *M*_0_, *Q*, *R*, and *T* are the coefficient of grain boundary mobility, activation energy, gas constant, and temperature, respectively.

**Figure 1:**
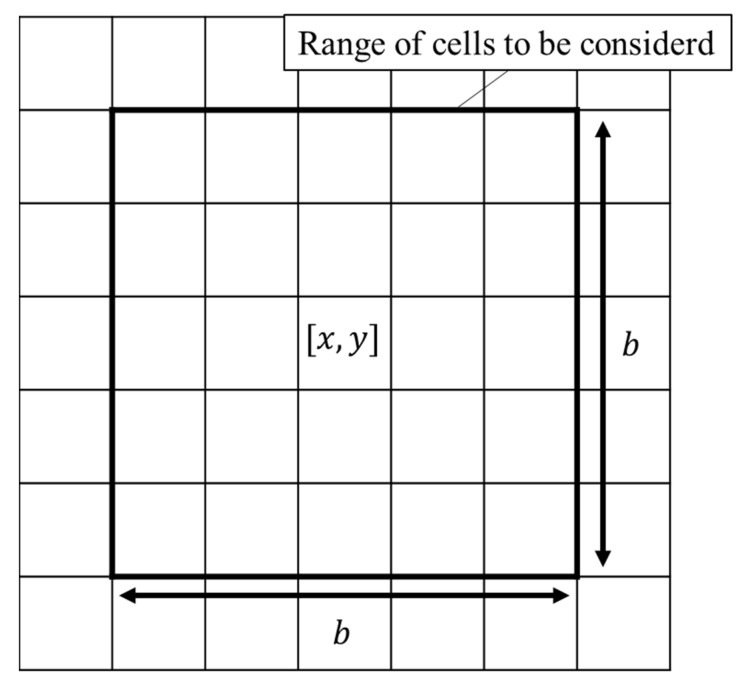
Schematic of the range of cells considered in the box count method.

Although the constants used in Equations (1) and (2) are well known, the curvature is not commonly calculated using the CA method. This is because the CA method considers space as discretely divided. According to the box count method [16], curvature radius *R_L_* can be expressed using the following equations:

(3a) $$R_{L}\left[x,y\right]=\frac{b^{3}}{n_{others}-b\times \frac{b-1}{2}},$$

(3b) $$n_{others}=\sum_{i,\;j=-\frac{b-1}{2}}^{\frac{b-1}{2}}\left({1-\delta }^{\left[x,\;y\right],\;\left[x+i,\;y+j\right]}\right),$$

(3c) $$\delta^{\left[x,\;y\right],\;\left[x+i,\;y+j\right]}=\left\{\begin{array}{c} 0\;if\;grain\left[x,y\right]\neq grain\left[x+i,y+j\right]\\ 1\;if\;grain\left[x,\;y\right][=grain[x+i,\;y+j] \end{array}\right.,$$

where b is the range of cells to be considered around cell x,y (Figure 1). For example, if *b* = 5, the curvature was calculated by considering the cells up to the second proximity. *δ*^[*x*,*y*][*x+i*,*x+j*]^ was 1 if cells *x*,*y* and *x + i*,*y + j* were different grains and 0 if they were the same.

This section describes the handling of crystal grains. In the CA and phase-field methods, each cell is assigned a unique grain number for identification. In this case, the misorientation between two cells was calculated using the absolute difference between the grain numbers divided by the maximum grain number, as shown in Equation (4) [15].

(4) $$\theta_{x+i,y+j}^{x,y}=\frac{\pi }{2}\frac{\left|grain\left[x,y\right]-grain\left[x+i,y+j\right]\right|}{G_{max}\;}\;\;\;\;\left[rad.\right],$$

where $\theta_{x+i,y+j}^{x,y}$ is the misorientation between cells *x*,*y* and *x + i*,*y + j*; grain[*x*,*y*] is the grain number of cell *x*,*y*; and *G_max_* is the maximum grain orientation number. However, the misorientation angles from Equation (4) differed from those of the real microstructure based on the two following points:

1. The maximum misorientation between two grains is 90°.
2. The misorientation distribution between two grains monotonically decreased toward the high-angle side.

Thus, we assigned a crystallographic orientation to each grain rather than a grain number. To simplify the calculation, we used a quaternion to represent the crystallographic orientation. The use of quaternions to represent grain orientations was proposed by Takahashi et al. [17].

**Figure 2:**
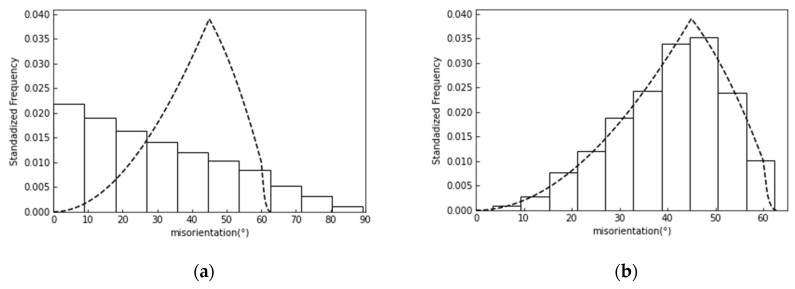
Distribution of grain boundary misorientation (dashed line represents ideal random misorientation distribution) (**a**) using grain numbers and (**b**) random crystallographic orientations.

The orientations of cubic crystals a and b were denoted as quaternions $q_{a}$ and $q_{b}$, respectively. Equation (5) was used to calculate the grain boundary misorientation as follows:

(5) $$\theta_{a,b}=2\sin^{-1}\left(\underset{1≦i≦24}{\min}\sqrt{1-{\left(q_{a}e_{i},\;q_{b}\right)}^{2}}\right)$$

where $e_{i}$ represents the 24 quaternion, which represents the symmetry operation of the cubic crystal. (**p**, **q**) represents the inner product of quaternions **p** and **q**. To obtain the grain boundary misorientation angles, the use of Euler angles requires more complicated operations and a higher computational cost than that of quaternions. Considering that the four real numbers of the quaternions can be treated equivalently, a random orientation can be easily produced by representing the four real numbers with uniform random numbers and normalizing them. Figure 2 compares two grain boundary misorientation angle distributions. Figure 2a shows the misorientation produced using the grain numbers, as proposed by Contieri et al. [15]. Figure 2b shows the misorientation obtained using random crystallographic orientations, as proposed in the present paper. The method used in the present study produced a grain boundary misorientation distribution that was closer to the theoretical distribution [18].

Next, the grain boundary energy of individual cells is discussed. Equation (6), reported by Read and Shockley, was applied to the grain boundary energy, and the orientation dependence of the grain boundary energy was incorporated [19,20]:

(6) $${Gb}_{x+i,y+j}^{x,y}=E_{0}\frac{\theta_{x+i,\;y+j}^{x,y}}{\theta_{m}}\;\left(1-\log\left(\frac{\theta_{x+i,y+j}^{x,y}}{\theta_{m}}\right)\right),$$

where ${Gb}_{x+i,y+j}^{x,y}$ is the grain boundary energy between cells *x*,*y* and *x + i*,*y + j*; *E*_0_ is the high-angle boundary energy; and *θ_m_* indicates the threshold angle at which the mobility was practically equal to that of the high-angle grain boundary. A *θ_m_* of 15° was used. The grain boundary energy, *σ*[*x*,*y*], of each cell was expressed as the sum of the grain boundary energies of the surrounding cells, which are Moore neighborhood cells (Equation (7)).

(7) $$\sigma \left[x,y\right]=\sum_{i,j=-1}^{1}{Gb}_{x+i,y+j}^{x,y}\left(1-\delta^{\left[x,y\right],[x+i,y+j]}\right).$$

The orientation dependence of *M* was introduced through Equation (8) [21]:

(8) $$M=M_{0}\exp\left(-\frac{Q}{RT}\right)\left(1-\exp\left(-5{\left(\frac{\theta }{\theta_{m}}\right)}^{4}\right)\right).$$

Considering the heterogeneity of the mobility, we devised a model that emphasized mobility by assigning higher mobility only to specific grains and performed simulations, as shown in Equation (9). This model assumed that only certain grains exhibited different orientations or coincident site lattice boundaries with the surrounding grains, resulting in a higher degree of mobility.

(9) $$M=\left\{\begin{array}{c} M_{0}\exp\left(-\frac{Q}{RT}\right)\left(1-\exp\left(-5{\left(\frac{\theta }{\theta_{m}}\right)}^{4}\right)\right)\;if\;not\;enhanced\;grain\\ {pM}_{0}\exp\left(-\frac{Q}{RT}\right)\left(1-\exp\left(-5{\left(\frac{\theta }{\theta_{m}}\right)}^{4}\right)\right)\;if\;enhanced\;grain \end{array}\right.,$$

where *p* is the coefficient of enhanced mobility.

Finally, the influence of precipitates on abnormal grain growth is discussed. According to Zener [22], the driving force (ΔG) of grain growth per unit volume can be obtained by introducing the pinning effect. Here, the 2D Zener equation was used.

(10) $$\Delta G\left[x,y\right]=\sigma \left[x,y\right]\left(\frac{1}{R_{L}\left[x,y\right]}-\frac{3f\left[x,y\right]}{2r\left[x,y\right]}\right),$$

where, *f* and *r* are the volume fraction and particle radius of the pinning particles, respectively.

Assuming that the pinning particles were unevenly distributed in the microstructure because of segregation or other effects, we confirmed that the grain growth behavior had a gradient when *f* was given a distribution, as shown in Equation (11).

(11) $$f\left[x,y\right]=f_{0}\left[\sin\left(\frac{2\pi my}{Y_{max}}\right)+1\right],$$

where *f*_0_ is the average volume fraction, *m* is an integer representing the degree of bias of the pinning particles, and *Y*_max_ is the number of cells in the *y*-direction.

The grain growth rate, *v*, was expressed as follows:

(12) $$v\left[x,\;y\right]=\Delta G\left[x,y\right]\times M\left[x,y\right].$$

**Figure 3:**
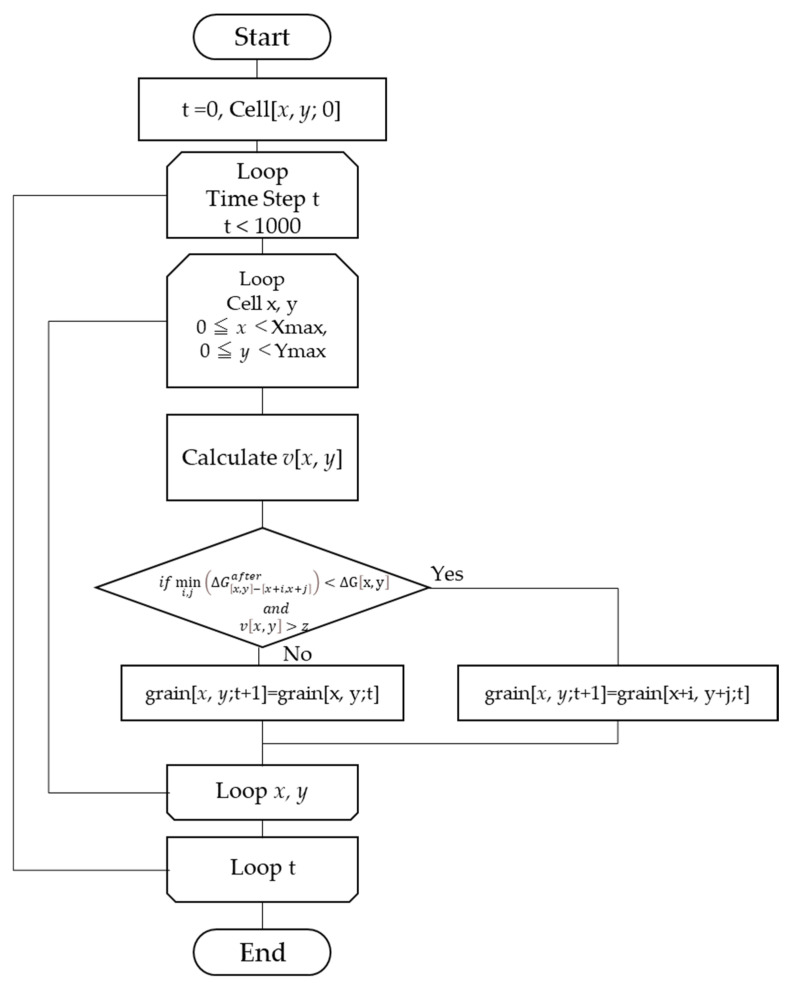
The overall flow of the CA simulation.

Here, the grain growth process was simulated following the rules shown in Equations (13a) and (13b). The rules state that if Δ*G* decreases and *v* is above a certain value (*z*, a random number), the grain will be in the same phase as the adjacent grains so that Δ*G* decreases to its lowest possible value.

(13a) $$grain\left[x,\;y\right]=grain\left[x+i,\;y+j\right]\;with\;\underset{i,j}{\min}\left({\Delta G}_{\left[x,y\right]-\left[x+i,\;y+j\right]}^{after}\right),$$

(13b) $$if\underset{i,j}{\min}\left({\Delta G}_{\left[x,y\right]-\left[x+i,x+j\right]}^{after}\right)<\Delta G\left[x,y\right]\;and\;v\left[x,y\right]>z,$$

where ${\Delta G}_{\left[x,y\right]-[x+i,x+j]}^{after}$ is the Δ*G* of cell *x*,*y* when only cell *x*,*y* was replaced by the grain of cell *x + i*,*y + j*. *z* is a uniform random number with the maximum value of *z*_max_; it was updated for each cell and step. The overall calculation is shown in Figure 3 as a flowchart.

The initial microstructure of grain growth was obtained using the CA method of phase transformation. Here, 1 px was calculated as 1 μm, and the initial average grain radius was 3.19 μm. Periodic boundary conditions were used as the boundary conditions.

The aforementioned simulations were performed using Python and C. Table 1 lists the calculation parameters.

## 3. Results and Discussions

**Figure 4:**
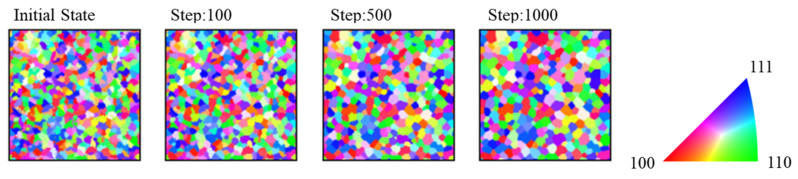
Microstructures during specific steps.

**Figure 5:**
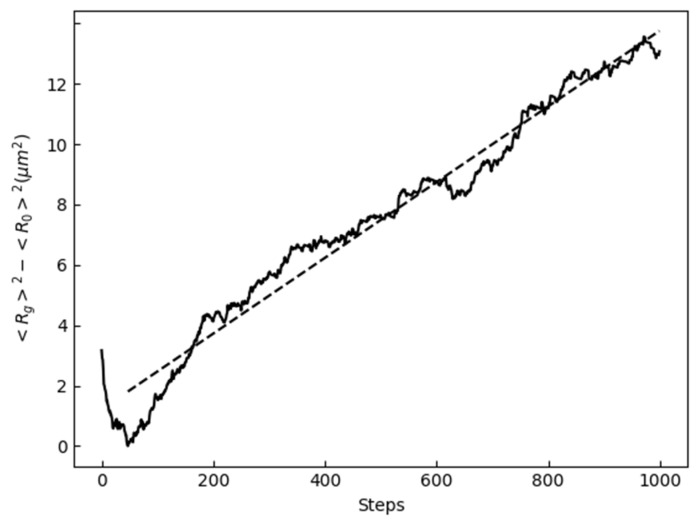
Time evolution of average grain radius.

The results of the simulation in which the mobility was not emphasized and precipitates were absent are discussed. Figure 4 shows the initial 100-, 500-, and 1000-step microstructures. The crystal orientations are presented in an inverse pole figure (IPF) map. Figure 5 shows the average grain radius at every step. The parabolic law was expressed by the following equation:

(14) $$<R_{g}{>}^{2}-<R_{0}{>}^{2}\propto t-t_{0},$$

where <*R*_g_> is the average grain radius, <*R*_0_> is the initial average grain radius, *t* is the time, and *t*_0_ is the reference time. The dashed line shown in Figure 5 is a straight line fitted using the least-squares method. Moreover, *t*_0_ is the time when $<R_{g}{>}^{2}$ was minimized from the initial state and grain growth began.

As shown in Figure 5, the average grain radius roughly followed the parabolic law, suggesting normal grain growth.

**Figure 6:**
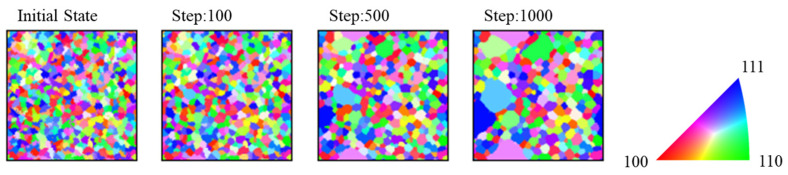
Microstructures during specific steps in the case of enhanced mobility.

Figure 6 shows the grain growth behavior when the mobility of the grains to be emphasized was 10 times that of others. The number of grains to be emphasized was 10. Abnormal grain growth was observed because of the enhanced mobility. The emphasis on mobility was considered equivalent to the effects of coincident site lattice boundaries or segregation. Olmsted et al. reported that the mobility of coincident site lattice boundaries considerably exceeded that of other grain boundaries [23]. A previous study also reported that grain boundary segregation reduced grain mobility [24].

**Figure 7:**
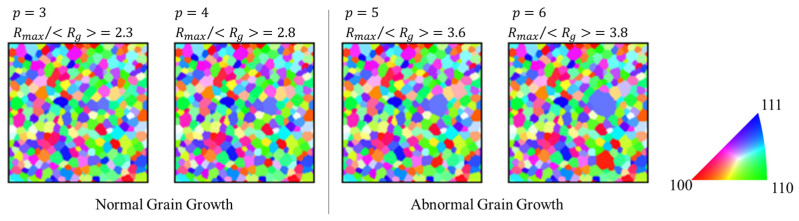
Effect of mobility coefficient *p* on the microstructure after 1000 steps.

The criterion for the occurrence of abnormal grain growth was based on Equation (15), which shows the ratio of the maximum grain radius, *R*_max_, to <*R*_g_> in the microstructure. When this ratio was more than 3, abnormal grain growth was considered to have occurred. Figure 7 shows an example of the microstructure at this time. Abnormal grain growth was considered to have occurred when the discriminant in Equation (15) was more than 3.

(15) $$\frac{R_{max}}{<R_{g}>}>3.$$

**Figure 8:**
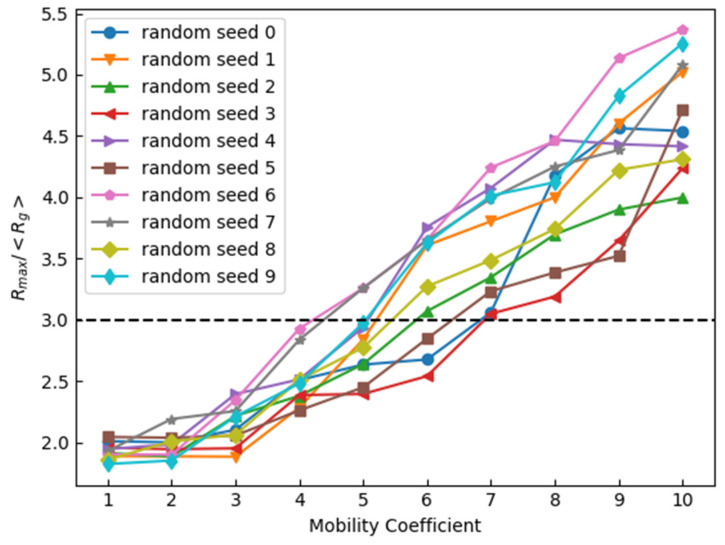
Effect of mobility coefficient on *R*_max_/<*R*_g_> after 1000 steps (Dotted line is abnormal grain growth criteria in Equation (15)).

**Figure 9:**
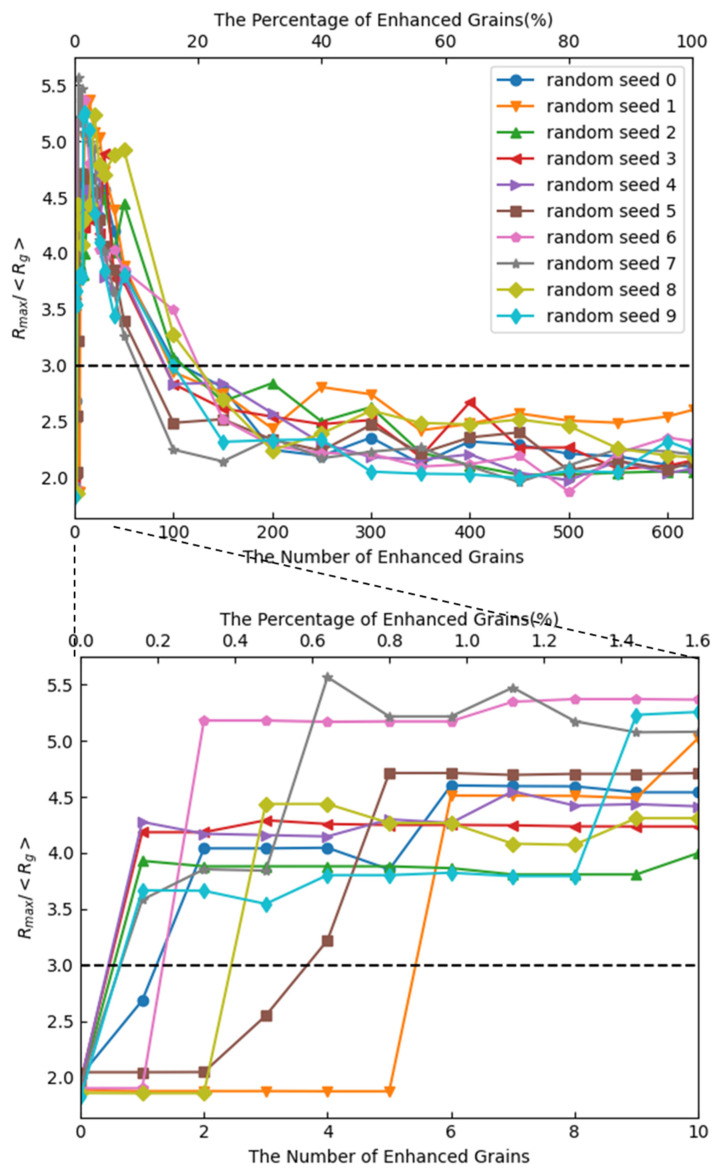
Effect of the number of enhanced grains on *R*_max_/<*R*_g_> after 1000 steps (Dotted line is abnormal grain growth criteria in Equation (15)).

Figure 8 shows the effect of the coefficient of enhanced mobility on the occurrence of abnormal grain growth. The x-axis represents the coefficient *p* of the enhanced mobility, and the y-axis represents the ratio of *R*_max_ to <*R*_g_> after 1000 steps. The number of grains to be emphasized is 10. Figure 9 shows the effect of the number of the grains to be emphasized on the occurrence of abnormal grain growth. The x-axis represents the number of the grains to be emphasized, and the y-axis represents the ratio of *R*_max_ to <*R*_g_> after 1000 steps. Here, 10 random number seeds were provided prior to the calculations. Ratios were obtained for each time the calculation was performed under the same conditions except for the 10 random number seeds. It should be noted that the results will depend on which random number seed is used, since the choice of the grain to be emphasized and the change in the cells at each time step depends on the random number.

Abnormal grain growth was observed when the mobility coefficient was 3 or higher, and it occurred in all cases in which the mobility coefficient was 7 or higher. Abnormal grain growth was observed when the number of grains emphasizing mobility became one but not when the percentage exceeded 20% of the number of all the grains in the initial state. This was because if the number of mobility-enhanced grains became extremely high, only the emphasized grains would grow normally.

**Figure 10:**
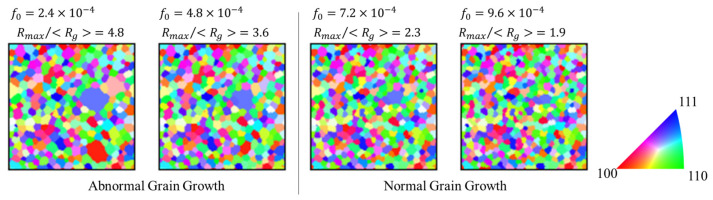
Effect of the volume fraction of precipitates on the microstructures after 1000 steps.

**Figure 11:**
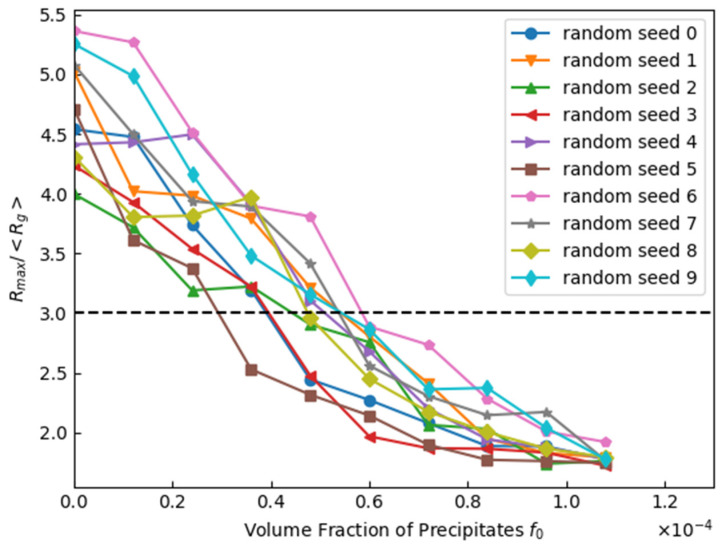
Effect of volume fraction of precipitates on *R*_max_/<*R*_g_> after 1000 steps (Dotted line is abnormal grain growth criteria in Equation (15)).

Figure 10 shows the effects of precipitates on the microstructure after 1000 steps. Figure 11 shows the effect of *f*_0_ on the discriminant in Equation (15). These results showed that the occurrence of abnormal grain growth can be suppressed by increasing the volume fraction of precipitates. The precipitate radius, *r*, was fixed at 5 nm regardless of position. The boundary mobility of the grains to be enhanced was set to 10 times, and the number of grains to be enhanced was set to 10. When no segregation occurred in the volume fraction of the precipitates, abnormal grain growth could be suppressed if the average volume fraction of the precipitates (*f*_0_) exceeded approximately 6.0 × 10^−5^.

Next, the correspondence between abnormal grain growth and Gladman’s law [25] is discussed. The critical precipitate radius r*, where abnormal grain growth occurs, is expressed in Equation (16):

(16) $$r*=\frac{6R_{0}f_{0}}{\pi }{\left(\frac{3}{2}-\frac{2}{Z}\right)}^{-1}$$

assuming that *Z* is the initial *R*_max_/<*R*_0_>, r* = 0.99 nm was calculated when the volume fraction of the pinning particles was set to *f*_0_ = 6.0 × 10^−5^, which suppressed abnormal grain growth. The precipitate particle radius used in this calculation was 5 nm, approximately five times larger than the value obtained from Gladman’s law (Equation (16)). However, a close-order value was obtained using this equation, which reproduced a behavior close to that of the actual structure.

**Figure 12:**
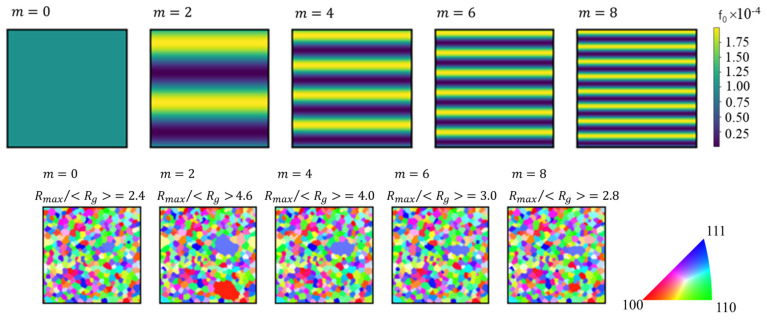
Distribution of f_0_ (upper row) and microstructures after 1000 steps (lower row).

Regarding the segregation of precipitates, Figure 12 shows the microstructure after 1000 steps when the precipitates were assumed to exist in a biased manner. Abnormal grain growth occurred at *m* < 2 (except when *m* = 0 without segregation). In addition, abnormal grain growth occurred in areas with a low volume fraction of precipitates. Thus, if the volume fraction of the precipitates was segregated, abnormal grain growth was more likely to occur. In particular, the larger the modulation of the segregation, the more likely abnormal grain growth was to occur. This indicated that the larger the segregation in the precipitate distribution, the less favorable it was for suppressing abnormal grain growth.

Summarily, we proposed a grain growth simulation model using factors, such as mobility and precipitate segregation, which have not been considered in previous studies, as far as we know. By considering these factors during the simulation, abnormal grain growth can be predicted. We believe that this study will facilitate microstructural control and material development.

## 4. Conclusions

This study attempted to reproduce abnormal grain growth and its suppression behavior by precipitates using the CA method with a crystallographic orientation. We arrived at the following conclusions:

1. Using the CA method, abnormal grain growth occurred when the degree of grain boundary mobility was emphasized and when the degree of emphasis was increased by a factor of three or more. This suggests that the degree of mobility of the grain boundaries may play an important role in abnormal grain growth.
2. Using the crystallographic orientation instead of grain numbers, it is possible to accurately reproduce the effects of grain boundary misorientations on energy and mobility.
3. Abnormal grain growth can be suppressed by the pinning effect of precipitates. However, if the concentration of the precipitates is uneven in the microstructure or if the pinning effect is weak in certain areas, it is difficult to suppress the abnormal grain growth.
4. The results indicate that the CA method can potentially reproduce abnormal grain growth. In addition, the findings would lead to reveal abnormal grain growth behavior during heat treatments, such as carburization.

## Figures and Tables

**Table 1:** Parameters for cellular automaton simulation.

Symbol	Value	Unit	Description
*Q*	1.82 × 10^6^ [23]	J/mol	Activation energy
*R*	8.314	J/(K·mol)·	Gas constant
*M*_0_	4.67 × 10^−6^ [23]	m^4^/(J·s)	Coefficient of grain boundary mobility
*E*_0_	0.75 [24]	J/m^3^	Grain boundary energy
*T*	1173	K	Temperature
*z*_max_	5 × 10^−7^	m/s	Maximum random number
*b*	7	-	Range of cells in the box count method
*X*_max_, *Y*_max_	150, 150	-	Size of calculation cells

## Data Availability

Data will be made available on request.
